# A comparison of surgical outcomes of perineal urethrostomy plus penile resection and perineal urethrostomy in twelve calves with perineal or prescrotal urethral dilatation

**Published:** 2013-10-13

**Authors:** M.A. Marzok, S.A. El-khodery

**Affiliations:** 1 *Department of Veterinary Surgery, Faculty of Veterinary Medicine, Kafrelsheikh University, Kafr El-Sheikh 33516, Egypt*; 2 *Department of Surgery and Gynecology, Faculty of Veterinary Medicine, University of Tripoli, Tripoli, Libya*; 3 *Department of Internal Medicine and Infectious Diseases, faculty of Veterinary Medicine, Mansoura University, Mansoura 35516, Egypt*; 4 *Department of Internal Medicine, Faculty of Veterinary Medicine, University of Tripoli, Tripoli, Libya*

**Keywords:** Male calves, Penile transaction, Perineal urethrostomy, Ultrasonography, Urethral dilatation

## Abstract

The clinical diagnosis, ultrasonographic findings, surgical management, outcome, and survival rate of perineal or prescrotal urethral dilatation in 12 male calves are described. All calves were crossbred and intact males. The most noticeable clinical presentations were perineal (n= 10) or prescrotal (n= 2) swellings and micturition problems. The main ultrasonographic findings were oval shaped dilatation of the urethra in all animals with dimensions of 40-75 X 30-62 mm. The calves with perineal urethral dilatation were treated by perineal urethrostomy (n= 4) and partial penile transection including the dilated urethra and urethral fistulation (n= 6). Prescrotal urethral dilatations were treated by penile transection proximal to the dilatation site (n= 2). Cystitis and stricture of the urethra were recorded postoperatively for two of the calves that underwent perineal urethrostomy. Nine animals were slaughtered at normal body weight approximately 6-8 months after the surgical treatment. Three animals were slaughtered after approximately three to four months, two of them having gained insufficient body weight. Our study shows that ultrasonography is a useful tool for the diagnosis of urethral dilatation in bovine calves. Our study also shows that the partial penile transection may be a suitable and satisfactory choice of surgical treatment for correcting the urethral dilatation in bovine calves.

## Introduction

The male urethra connects the urinary bladder to the glans of the penis and consists of pelvic and extra pelvic parts (Sisson and Grossman, 1975). Urethral dilatation is an uncommon clinical problem that is observed in male animals and humans.

In calves, congenital urethral dilatation (megalourethra) and developmental urethral dilatation in the perineal region has been reported only in isolated cases (Tharp and Venzke, 1954; Johnson *et al.*, 1980; Gasthyus *et al.*, 1996; Geccelep and Alkan, 2000). The condition occurs sporadically and is observed during the first week to several months of age (Anderson *et al.*, 1993; Sedeek and Bakr, 2009).

The etiology of megalourethra is unknown in either animals or humans (Kester *et al.*, 1990). Urethral dilatation in juvenile male cattle is a rare phenomenon probably related to a transient urethral obstruction or to a developmental abnormality (Gasthyus *et al.*, 1996).

Clinical signs of urethral dilatation include the presence of a fluctuant, firm, irreducible and painless swelling in the perineal region with dysuria. Differential diagnoses include pathological perineal, retroflexion and hernia of the urinary bladder, rupture of the urethra and pathological urethral diverticulum or dilatation (Weaver *et al.*, 1992). The condition is usually associated with urethritis and/or cystitis as a result of accumulation of urine in the swelling for a prolonged period (Parsons *et al.*, 1998).

Treatment of urethral dilatation is directed towards surgical correction, when possible. Surgical treatment involves either perineal urethrostomy or excision of the urethral dilatation (Tharp and Venzke, 1954; Gasthyus *et al.*, 1996; Geccelep and Alkan, 2000; Sedeek and Bakr, 2009).

However, due to the rarity of the condition, clinical reports on urethral dilatation in bovine calves are scarcely reported in the literature. To our knowledge, there are no reports describing urethral dilatation in the prescrotal region of bovine calves. Therefore, the purpose of this article is to describe the clinical diagnosis, ultrasonographic findings, surgical management, outcome, and survival rate in 12 male calves presented with urethral dilatation either in the perineal or the prescrotal regions.

## Materials and Methods

### Patient selection

Twelve calves were presented to the veterinary teaching hospital of Kafrelsheikh University, Egypt, between 2006 and 2011 with the chief complaint of penile urethral dilatation.

**Fig. 1:**
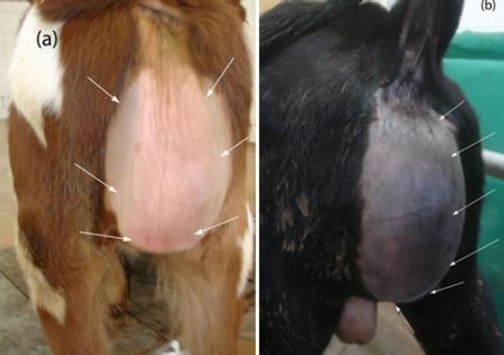
(a) Caudal and (b) lateral view of the perineum of two animals, showing the clinical presentation of an urethral dilatation in the perineal area (white arrows).

**Fig. 2:**
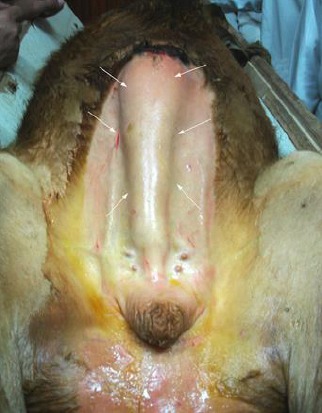
Urethral dilatation in the prescrotal area (white arrows).

All cases were found to have the dilatation at the perineal region (n= 10) or the prescrotal region (n= 2) (Figures 1 and 2).

Calves with perineal urethral dilatation were randomly classified into two groups; group-I (n= 4) and group-II (n= 6), which were then treated by perineal urethrostomy or partial penile transection (including the dilated urethra) and urethral fistulation, respectively. While, calves in group-III (n= 2) with prescrotal urethral dilatation were treated by penile transection (amputation).

Data collected included signalment, anamnesis, physical and clinical examination findings including ultrasonographic observations, surgical technique (duration, cost, severity of bleeding) and complications, body gain and surgical outcome.

### Clinical diagnosis

Ultrasound examination for the urinary bladder and the urethra was carried out using a real time; B-mode diagnostic ultrasound scanner (Pie-Data Medical Company, Maastricht, The Netherlands, 100 LC) equipped with linear array 6/8 MHz transducer and 3.5/5.0 MHz sector transducers.

Transrectal examination of the urinary bladder and the urethra was performed for all presented cases in a standing position without sedation. The penile urethra was also examined via transcutaneous ultrasonography of the perineal and prescrotal areas. Transabdominal ultrasound examination of the right kidney was also performed (Braun, 1991), but no abnormal findings were visualized.

Diagnosis was confirmed with aseptic fine needle aspiration of the swellings using a 16 gauge hypodermic needle attached to a 50 ml syringe. Blood samples were collected from each animal for haematology and biochemistry.

### Surgical procedures

**Fig. 3:**
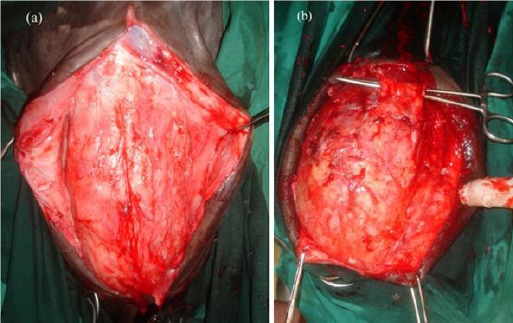
(a) Intraoperative view of the dissected dilated urethra; (b) Identification of the retractor penis muscle.

**Fig. 4:**
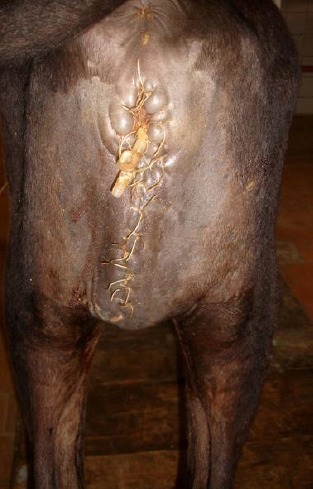
One week post-operation in a calf which underwent partial penile transection.

All animals were treated surgically while standing (Figures 1, 2, 3, and 4). Epidural analgesia was induced by injection of 2-3ml of lidocaine 2% (Debocaine 2%; El-nasr pharm, Egypt) into the 1st intercoccygeal vertebral spaces.

In 4 animals, 0.05 mg/kg xylazine hydrochloride (Xylaject 2%; Adwia, Egypt), diluted in 5 ml of sterile saline, was administered into the epidural space to induce adequate analgesia of the perineal region. Surgical repair was conducted after aseptic preparation of the perineal area.

### Group I

Urethrostomy was performed through a mid-line skin incision at the distal end of the swelling. The enlarged urethral cavity was exposed by blunt dissection. The wall of the urethra was incised and the mucosa of the dilated urethra was sutured to the skin with single interrupted sutures (Supramid #1 or 2).

Finally, the urethral cavity was flushed with physiological saline to remove any debris. Postoperative treatment included IM injection of 7 mg/kg long acting amoxicillin (Amoxinject LA, Bremer Pharma GMBH, Germany), and daily cleaning and/or flushing of the urethral orifice with povidone iodine solution.

### Group II

A 25-30 cm vertical midline skin incision was made, extending from 5 cm ventral to the anus to 10 cm proximal to the scrotum, after which blunt dissection was used to separate the firm fibrous walled and hollow structures from the body wall. The retractor penis muscles, identified caudal to the perineal sac (Figure 3), were resected from the region over the perineal swelling.

The sac was firmly attached to the underlying musculature cranially, and muscle fibers of the ischiocavernous were identified passing obliquely from the proximal part of the sac onto the caudal ischial musculature and fascia.

The perineal sac was then transected at its origin and termination, and dissection separated its attachments to the perineal raphè. The penis distal to and contiguous with the sac was ligated with single encircling suture of # 2 supramid at two points proximal to the sigmoid flexure and was transected between these sutures. The proximal attachment of the sac was transected 2-5 cm distal to the caudal portion of the pelvic urethra. The edges of the sac were sutured to the skin in a single interrupted pattern using # 1 supramid, completing the perineal urethrostomy procedure.

A 10-12 gauge urinary catheter was passed into the urinary bladder through this orifice and fixed laterally to skin in a single interrupted pattern using # 0 supramid. After emptying the urinary bladder it was irrigated several times with a warm solution of 0.9% saline. To obliterate the dead space below the swelling, the perineal incision was closed using subcutaneous appositional simple sutures of #1chromic catgut and skin sutures of # 2supramid placed in a simple pattern (Figure 4).

All animals were given ethamsylate 250 mg IV (Dicynone®, Minapharm Pharmaceutical Co., Egypt) 20 minutes prior to the surgery to control bleeding from the surgical site, Ringer’s solution (a total of 3 - 4 L IV) during surgery, and long acting amoxicillin 7 mg/kg IM (Amoxinject LA, Bremer Pharma GMBH, Germany) and 2.2 mg/kg IV flunixin meglumine (Flunixin Injection, Norbrook Laboratories Limited, NEWRY, Co. down, Northen Ireland) after completion of the surgery. The resected specimens were fixed in 10% formalin and submitted for histological examination. All calves were discharged the same day with instructions to the owner to continue anti-inflammatory treatment for a further 4 days and to have the sutures removed on day 14 under veterinary supervision.

### Group III

A midline incision, 10-15 cm long was made in the perineal region ventral to the anus and extended ventrally toward the scrotum. The incision was deepened through the subcutaneous and dense connective tissue between the semimembranosus muscles until the paired retractor penis muscles and penis were identified. The penis was then grasped, traced caudally and dorsally and dissected from the surrounding tissue.

Once the penis was exteriorized, the retractor penis muscles were ligated and transected as far proximally as possible. The dorsal vessels of the penis were also bluntly dissected and preserved. The penis was transected about 5 cm distal to the dorsal part of the skin incision. The distal stump of the penis was reduced with intact dorsal vessels, meanwhile the proximal penile stump was fixed to the skin using horizontal mattress suture surrounding and ligating the corpus cavernosum.

Finally the incision above and below the penile stump was closed with continuous suture pattern using # 2supramid. Aftercare was made in the same manner as in group (I).

### Follow-up

Follow-up information was obtained via telephone contact with veterinarians and owners at least 6 months after surgery.

## Results

### Animals

All the calves were crossbred intact males. The average age at presentation was 9.1± 1.69 months (range: 7 months to 12 months). Average body weight was 98.3± 21.46 kg (range, 70–130 kg).

### History and clinical evaluation

The history of 8 (66.6%) cases indicated that the swellings were noticed by the owner for a period of 4 days to 6 weeks. The remaining cases (n= 4) were recently purchased from the market; consequently, the time of disease onset was unknown. Stranguria has been observed in all cases.

Clinical examination revealed that all affected calves were alert and with a fair body condition, with normal rectal temperature, pulse, and respiration. Firm, irreducible, and fluctuant swellings (10-20 cm in diameter) were palpated either in the mid-perineal region ventral to the anus extending ventrally to the scrotum or in the ventral surface of the penile urethra 2 cm caudal to the preputial orifice and extending caudally towards but not involving the scrotum.

The calves had no signs of discomfort during micturition or palpation of the enlargement. Urine dripped intermittently from the prepuce and palpation of the swelling caused urine to dribble excessively from the preputial orifice.

Stimulation of micturition caused an obvious distension of the swelling that was followed by continuous passive dribbling of turbid white yellow colored urine. In 4 (33.3%) cases, mucous strands were seen mixed with the urine. Ultrasound examination per rectum revealed moderate distension of the urinary bladder.

The main hematological and biochemical values were within normal physiological limits (despite masses of white blood cells in the aspirated fluid). Aseptic fine needle aspiration of the swellings yielded a large volume (200-400 ml) of clear yellow fluid that had ammoniacal or urineferous odor. Masses of white blood cells, few red cells, a few cocci, calcium phosphate crystals, and strands of mucous were observed in the cytological examination of the urine.

### Ultrasonographic findings

Ultrasonography revealed that the urinary bladder was both intra-pelvic and intra-abdominal with the neck being located at the caudal border of the ischium. In eight cases, the bladder and bladder wall thickness were normal.

**Fig. 5:**
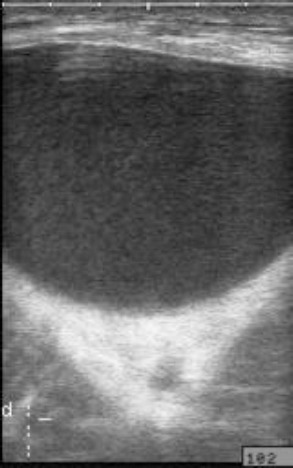
Ultrasound image of a calf with a dilated penile urethra containing anechoic and hypoechoic materials. Notice the thickened urethral wall.

**Fig. 6:**
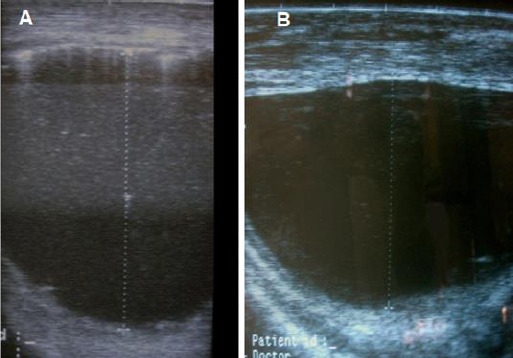
Ultrasound image of a calf with a dilated penile urethra. (A) The caudo-cranial diameter is 75 mm and (B) latero-lateral diameter is 32 mm. Notice the thickened urethral wall.

Cystitis was diagnosed in 4 (33.3%) cases, in which the wall of the bladder was thickened, and the urine was turbid. The main ultrasonographic findings were oval shaped dilatation of the urethra in all animals with dimensions of 40-75 X 30-62 mm. The contents appeared anechoic and hypoechoic in every direction (Figures 5 and 6).

**Fig. 7:**
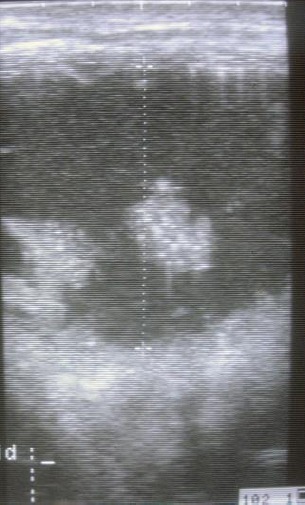
Dilated urethra containing hypoechoic and echoic material consistent with fibrin strands.

The echogenic pattern of the urethra was interrupted by swirling points due to the presence of numerous erythrocytes in the urine - salbuminous appearance of urine - not caused by erythrocytes. Numerous irregular hyperechoic masses, consistent with fibrin strands, were visualized floating within the fluid in 4 cases (4/12) (Figure 7).

**Fig. 8:**
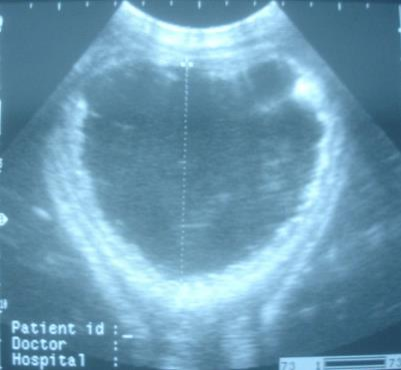
Sonogram of dilated penile urethra (the caudo-cranial diameter is 77 mm) obtained from a calf with necrotizing urethritis. Notice the marked thickening of the urethral wall and irregular mucosal surface.

A marked thickening of the urethral wall (urethritis) was observed in all animals. The average urethral wall thickness was 1.04±0.27 mm. In three animals (3/12), an irregular mucosal surface was also noted (Figure 8). In all cases, ultrasound examination of the right kindney showed no abnormal findings.

### Surgical treatment

Primary repair of urethral dilatation was performed by perineal urethrostomy (n= 4), partial penile transaction, including the dilated urethra and urethral fistulation (n= 6) and penile transaction distal to the dilatation site (n= 2).

**Fig. 9:**
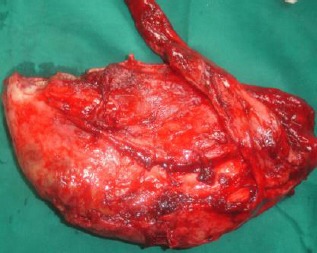
The resected penile urethra showing muscle and adipose tissue adhered to the urethral wall.

Average duration of surgery was 31.75 ± 6.23 minutes (range, 20–45 minutes), 88, 00 ± 13.03 (range, 70–100 minutes), and 45.00 ±7.07 (range, 40–50 minutes), respectively. The resected specimens were dilated, empty pouch, narrowing at both ends of its 25-30 cm length (Figure 9).

**Fig. 10:**
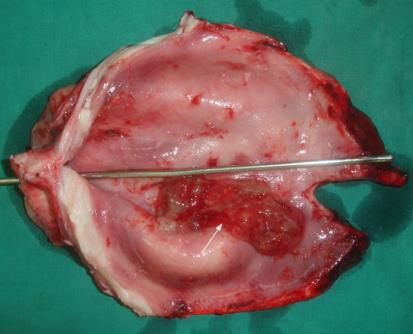
The excised penile urethra showing the narrow and dilated segments of the urethra. Accumulation of mucous threads were observed inside the urethral dilatation (white arrow).

**Fig. 11:**
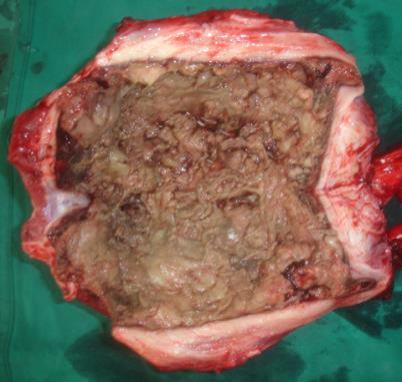
The excised penile urethra showing a necrotic mucosal surface.

An accumulation of urine mixed with blood, debris, mucous threads or pus was observed inside the urethral dilatation during surgery and post resection (Figure 10). In three animals, the urethral mucosa was almost necrotic and a pseudodiphtheritic membrane was present (Figure 11).

From a technical point of view, the surgical interventions were quickly and easily performed in both the urethrostomy and penile transection groups. Bleeding and disruption of the soft tissue were extensive in the partial penile resection technique when compared to the other methods. With respect to the cost of surgery and aftercare, it was found that the cost of urethrostomy was less than the cost of penile transection; meanwhile the cost of its aftercare was twice that of the penile transection procedures.

### Outcome

Follow up of all cases was done via phone calls to the referred veterinarian. The recovery period ranged from 10 to 18 days (average, 12.9 ±3.2 days). The calves were discharged on the same day. No postoperative complications were observed in groups II and III. Cystitis and stricture of the urethra were recorded for two of the calves that underwent perineal urethrostomy. These calves were readmitted to the clinic 2 months after discharge and recovered following widening of the urethrostomy fistula.

Ten animals were slaughtered at normal body weights approximately 6-8 months later. Two animals (group I) were slaughtered after approximately three to four months because of an inadequate weight gains.

## Discussion

Perineal urethral dilatations in calves encountered in the current investigation were similar to those described by previous workers (Johnson *et al.*, 1980; Weaver *et al.*, 1992; Anderson *et al.*, 1993; Gasthyus *et al.*, 1996; Geccelep and Alkan, 2000; Sedeek and Bakr, 2009). The occurrence of this lesion in the prescrotal urethra appears to be the first report of this case in calves.

Urethral dilatation is observed at the proximal perineum in heifers and adults, and emerges as a result of transient urethral obstruction, or much less frequently due to bacterial urethritis of the lower urinary tract (Karrass *et al.*, 1992; Gasthyus *et al.*, 1996). Congenital defects of the urinary tract are not common in farm animals (Dennis and Leipold, 1979).

Urethral dilatation is a rare congenital or developmental malformation of the urethra in domestic mammals. Megalourethra is uncommon in male human neonates and is characterized by a dilatation of the penile urethra, a poor urine stream, and infection of the distal urethra without the evidence of urethral stricture (Nesbitt, 1954; Schrom *et al.*, 1981). Megalourethra has been reported in a male Charolais calf (Weaver *et al.*, 1992).

The etiology of megalourethra is currently unknown in both animals and human beings. Urethral dilatation related to transient urethral obstruction or developmental abnormality has been reported in 7 juvenile male cattle (Gasthyus *et al.*, 1996). The later observation of urethral swelling by the owners in this study, suggests that the condition was developmental.

All the animals were crossbred bulls. The preponderance of this breed was probably due to the popularity of these animals in Egypt. Urethral dilatation has been reported in Holstein-Friesian (Tharp and Venzke, 1954), Hereford (Anderson *et al.*, 1993), Charolais (Weaver *et al.*, 1992) and double-muscled Belgian blue bulls (Gasthyus *et al.*, 1996).

Because most of the animals were reported to have had micturition problems and debris, mucous threads and/or pus were often found during surgery, the urethral dilatation might have been due to an earlier partial urethral obstruction distal to the dilated area. As a result of repetitive partial obstruction a gradual increase in the pressure in the urethral canal can occur, leading to a proximal dilatation (Gasthyus *et al.*, 1996). Dilatation of the urethra is accompanied by urinary stasis which results in bacterial urethritis from ascending infections. Making a precise diagnosis in the field is of great interest as it permits rapid decision making regarding treatment options and avoids wasteful supportive treatments in the case of a low-value animal.

Centesis of the dilated mass under aseptic conditions is often a simple but effective diagnostic aid. In this study the material obtained by centesis of the urethral swelling was urine, usually mixed with blood and other debris. The debris indicated the presence of inflammation and/or infection of the urinary tract. Urinary tract diagnostic imaging techniques such as plain radiography, contrast urethrogram, intravenous urogram and ultrasonography, can be used to confirm the involvement of the urinary tract (Karrass *et al.*, 1992).

The margins of the perineal swelling were easily determined by ultrasonography, also enabling the diagnosis of its contents.

Braun (1993) mentioned that it is rarely necessary to anaesthetize animals for ultrasound examination. The findings of the present study match the previous suggestions because ultrasound examination was done for all cases with the animals in standing position and without sedation. Ultrasound measurements of the wall thickness of the urinary bladder and urethra were claimed to be useful method to determine the increase in the wall thickness in cases of cystitis and urethritis (Hoque *et al.*, 2002).

In present study, ultrasound examination was helpful in measuring the thickness of the urinary bladder and the urethral wall in four cases of cystitis and in all cases of urethritis. Calves with urethral dilatation frequently have common history and physical examination findings. The clinical findings in the present study were in agreement with those of other reports (Tharp and Venzke, 1954; Johnson *et al.*, 1980; Anderson *et al.*, 1993; Gasthyus *et al.*, 1996; Sedeek and Bakr, 2009).

The main observation in all animals was a fluctuant swelling in the perineal or prescrotal regions. The differential diagnoses of perineal and prescrotal swelling include haematoma or abscess; retroflexion of the urinary bladder; congenital urethral abnormality; pathological urethral diverticulum or dilatation; and urethral rupture (Weaver *et al.*, 1992). Centesis of the fluctuant masses eliminated the first two possibilities. Retroflexion of the bladder was considered unlikely for anatomical reasons and also because it was likely to result in complete retention of urine and severe tenesmus as seen in perineal hernia in the canine males (Canfield and Bellinger, 1985). Retroflexion was definitively eliminated as a differential diagnosis when tranrectal ultrasonography revealed the normal position of the urinary bladder in the pelvic and abdominal cavities.

Ultrasonographic examination revealed an intact urethra in all cases, excluding urethral rupture. Although general anesthesia is commonly used in cattle in hospital settings, there are some risks associated with its use (Edmondson, 2008). Local or regional anesthesia is safe and effective, and is still the most desirable procedure in many situations. In the present study, all the surgical interventions were performed in the standing animal under the influence of epidural analgesia.

The epidural administration of alpha-2 agonists such as xylazine hydrochloride (St Jean *et al.*, 1990) was an effective analgesic technique for the surgeries described. Surgery has been suggested by many authors as the standard treatment for urethral dilatation in calves (Karrass *et al.*, 1992; Gasthyus *et al.*, 1996). Conservative treatment described in one report (Anderson *et al.*, 1993) was hard to justify in this study because of the problems with micturition. Different surgical techniques including urethrotomy or partial penile transection have been reported to be successful in the management of urethral dilatation in calves. The reported success rate of surgical treatment by urethrostomy varied from 37.5 to 100% (Tharp and Venzke, 1954; Gasthyus *et al.*, 1996; Sedeek and Bakr, 2009). Whereas the reported success rate of partial penile transection was 100% (Weaver *et al.*, 1992; Sedeek and Bakr, 2009). Similar findings were shown in this investigation where the success rates of urethrostomy and partial penile transection was 50% and 100%, respectively.

Our study suggests that the urethrostomy technique takes shorter time than the partial penile amputation technique. This is likely to be related to the complexity of the different surgical procedures. Severe bleeding in partial penile transaction, haemorrhage control may explain the prolonged surgical times, despite pre-surgical injection of a haemostatic drug. Subsequently, this technique is potentially more expensive than urethrostomy, although at our clinic the price for each surgery was similar. The observed complications in the urethrostomy group agree with that reported previously (Anderson *et al.*, 1993; Gasthyus *et al.*, 1996). However, no complications were observed in the penile transection groups.

The higher incidence of cystitis in urethrostomy group might be related to pooling of urine caudoventral to the ventral commisure of the urethrostomy opening. Stricture of the urethrostomy opening is a common complication of urethrostomy that may require re-widening multiple times for establishment of a stoma, but generally it has limited success rate (Haven *et al.*, 1993; Van Metre *et al.*, 1996). In the present study, stricture was recorded in two calves and may be due to many factors including: the invasive nature of the procedure, urethral scarring, and predisposition for re-obstruction (Harari, 2003), pooling of urine caudoventral to the ventral commisure of the urethrostomy with continuous irritation of the skin around the stoma and formation of granulation tissue; and finally the direct contact of the two edges of the wound via pressure of the medial aspect of the thighs.

On the contrary, partial penile transaction was not associated with stenosis despite being more invasive. Decreased stenosis may be due to the higher position of the created stoma (directly under anus) and the fixation of a urinary catheter at this position that prevented direct contact of wound edges and accumulation of urine at the ventral commisure.

## Conclusions

Ultrasound examination was found to be a useful non-invasive diagnostic technique for cases of urethral dilatation in bovine calves. This is the first report that describes the prescrotal urethral dilatations in calves. Partial penile transection can be an effective and satisfactory surgical treatment for urethral dilatation in bovine calves. Generally this technique minimizes the risk of postoperative complications and yields improved outcomes, compared with urethrostomy.
